# Time to focus on outcome assessment tools for childhood vasculitis

**DOI:** 10.1186/1546-0096-9-29

**Published:** 2011-09-26

**Authors:** Erkan Demirkaya, Raashid Luqmani, Nuray Aktay Ayaz, Abdulbaki Karaoglu, Seza Ozen

**Affiliations:** 1 Erkan Demirkaya, Gulhane Military Medical Faculty, School of Medicine, Division of Pediatric Nephrology and Rheumatology, 06018 Etlik, Ankara, Turkey; 2 Raashid Luqmani, Rheumatology Department, NIHR Biomedical Research Unit, Botnar Research Centre, Oxford University, Oxford, UK; 3 Nuray Aktay Ayaz, SB Istanbul Bakırköy Maternity and Childrens Education and Research Hospital, Division of Pediatric Rheumatology, Istanbul, Turkey; 4 Abdulbaki Karaoglu, Gulhane Military Medical Faculty, School of Medicine, Department of Paediatrics, 06018 Etlik, Ankara, Turkey; 5 Seza Ozen, Hacettepe University Medical Faculty, School of Medicine, Division of Pediatric Nephrology and Rheumatology, 06100 Sihhiye, Ankara, Turkey

**Keywords:** Childhood vasculitis, Birmingham vasculitis activity score, disease extent index, Pediatric vasculitis damage index, outcome measurement, disease activity assessment

## Abstract

Childhood systemic vasculitides are a group of rare diseases with multi-organ involvement and potentially devastating consequences. After establishment of new classification criteria (Ankara consensus conference in 2008), it is now time to establish measures for proper definition of activity and damage in childhood primary vasculitis. By comparison to adult vasculitis, there is no consensus for indices of activity and damage assessment in childhood vasculitis. Assessment of disease activity is likely to become a major area of interest in pediatric rheumatology in the near future. After defining the classification criteria for primary systemic childhood vasculitis, the next step was to perform a validation study using the original Birmingham vasculitis activity score as well as the disease extent index to measure disease activity in childhood vasculitis. Presently, there are efforts in place to develop a pediatric vasculitis activity score. This paper reviews the current understanding about the assessment tools (i.e., clinical features, laboratory tests, radiologic assessments, etc.) widely used for evaluation of the disease activity and damage status of the children with vasculitis.

## Background

The primary systemic vasculitides (PSV) in children encompass a group of rare diseases that are characterized by the inflammation of blood vessels [1]. Although Henoch-Schonlein purpura (HSP) and Kawasaki disease (KD) are quite common forms of vasculitis, polyarteritis nodosa (PAN), Wegener's granulomatosis (WG) and Takayasu arteritis (TA) are diagnosed less commonly in children. Because PAN, WG, and TA affect many body systems, they have a wide range of clinical presentation and, if left untreated, follow a chronic relapsing course with high mortality and morbidity. With the wider use of immunosuppressive agents, both mortality and morbidity rates have been declining in childhood vasculitides [2].

The majority of children with HSP experience a full and uneventful recovery. Primary outcome measures for HSP are renal survival and presence of urinary abnormalities or hypertension. For unselected patients with HSP, the risk of end stage renal disease (ESRD) is reportedly very low, between 1.5% and 3% [3]. Recently Butani et al. demonstrated that even in an unselected population of patients who had HSP glomerulonephritis in childhood (n = 65), a significant proportion [n = 12 (23%)] progress to ESRD on long-term follow-up (20 years). They reported that the only factor associated with development of ESRD was the use of cytotoxic agents [4].

Prognosis for individuals with PAN varies with a better outcome in children compared to adult onset disease where the mortality rate can be as high as 20-30% despite aggressive therapy [5]. Brogan et al. reported the mortality rate for PAN in children as approximately 10% [6].

Despite the new immunosuppressive and immunomodulatory therapies, WG, microscopic polyangiitis (MPA) and Churg-Strauss syndrome (CSS) still carry considerable morbidity and mortality mainly due to progressive renal failure or aggressive respiratory involvement. In a recent report by Cabral et al., significant renal involvement was found in 27 out of reported 117 children with WG (41.5%). In the same report, severe respiratory involvement requiring continuous oxygen therapy or mechanical ventilation was observed in 19% and 11%, respectively [7]. Akikusa et al. reported that significant long-term renal impairment still occurs in 17-40% of children with WG [8]. The mortality rate for pediatric WG was 12% in another pediatric series [9]. The prognosis for MPA is similar to that of PAN in terms of mortality, and 18% for CSS in children according to the most recent series [10,11].

As a result of increased survival and well being in these patients, disease classification and assessment have emerged as important considerations. Established classification criteria are necessary in order to proceed with the development of future epidemiological studies and clinical trials in PSV.

In 1990, the American College of Rheumatology (ACR) grouped systemic vasculitides and proposed classification criteria for adult patients [12-15]. However, this system overlooked the presence of the antineutrophil cytoplasmic antibody (ANCA) in some forms of vasculitis, and completely overlooked microscopic polyangiitis (MPA). At the 1994 Chapel Hill Consensus Conference, vasculitis definitions were published, but these definitions were not designed to classify the vasculitides [16]. Currently, a working group supported by the European League Against Rheumatism (EULAR) and the ACR is revising the definitions and classification criteria for adult vasculitis (http://www.clinicaltrials.gov, NCT01066208). Though children and adults share many features of these rare disorders, ACR classification criteria developed for adults were not appropriate for use in children. The patient groups upon which the ACR had based the vasculitis classification criteria did not include children [17]. In 2005, the European League Against Rheumatism/Pediatric Rheumatology European Society (EULAR/PReS) vasculitis working group proposed new classification criteria for children with vasculitis predominantly according to vessel size, as follows: small [HSP, childhood Wegener's granulomatosis (c-WG)], medium [KD and childhood polyarteritis nodosa (c-PAN)], and large [childhood Takayasu arteritis, (c-TA)] [18]. The criteria were validated for use by EULAR, PReS and Paediatric Rheumatology International Trials Organisation (PRINTO). At the 2008 Ankara Consensus Conference, the final consensus was reached and the reports were published in 2010 for classification of childhood vasculitis (Table 1) [18-20]. Highly sensitive and specific validated definitions for HSP, c-PAN, c-WG and c-TA were established. It was clear that with the aid of this classification scheme, it would become easier to group patients, to conduct multicenter collaborative clinical trials and studies, and to manage individual patients.

**Table 1:** Classification of Childhood Vasculitis by Özen et al.

Predominantly large-vessel vasculitis	Predominantly medium-sized vessel vasculitis	Predominantly small-vessel vasculitis	Other vasculitides
Takayasu arteritis	Childhood polyarteritis nodosa (c-PAN)	Granulomatous: Wegener granulomatosis, Churg-Strauss syndrome	Behçet Disease

	Cutaneous polyarteritis	Non-granulomatous: Henoch-Scönlein purpura, Microspcopic polyangiitis, Hypocomplementemic urticarial vasculitis	Vasculitis secondary to infection (including Hep B associated PAN), malignancies and drugs, including hypersensitivity vasculitis

	Kawasaki Disease		Vasculitis associated with connective tissue diseases

			Isolated vasculitis of the central nervous system

			Cogan syndrome

			Unclassified

The site of involvement, the size of the affected vessel, and the extent of involvement defne the clinical features of vasculitis in children. Unlike the situation in adult vasculitis, HSP and KD are the most frequently encountered vasculitis sub-groups in children [21,22]. They both usually have an acute onset and a self-limiting course. But similar to adults, although very rare, c-PAN, c-WG and c-TA have an insidious course in children. Histopathological evaluation or imaging techniques such as angiography are gold standards for their diagnoses as in adults.

If left untreated, the PSV have a higher rate of morbidity and mortality. Although new treatment regimens have reduced these rates, complications due to multi-organ involvement of the vasculitis or the medications used are still an important problem.

Since new classification tools are now available to pediatricians as outlined above, it is time to consider the development of outcome assessment tools for childhood vasculitides. Assessment of disease activity is likely to become a new and crucial focus of research in pediatric rheumatology. In the last decade, disease activity indices have been developed for juvenile idiopathic arthritis (JIA), juvenile dermatomyositis, systemic lupus erythematosus (SLE); however, there have been no disease assessment tools defined and validated yet specifically for PSV [23-27].

## General Approach to Outcome in Pediatric Systemic Vasculitis

Physical examination, laboratory assessment, histopathological evaluations and radiological investigations are important tools both in the diagnosis and assessment of disease activity in PSV [28,29].

### Physical examination

Children usually have fever, malaise, weight loss, myalgias and rashes, but these symptoms are non-specific for vasculitis. It is clear that while thorough history-taking and a careful physical examination are crucial for the correct diagnosis, the subsequent assessments over time of disease activity, disease progression, and need for effective treatment protocols have a similar important role in the child's outcome. Although remission is usually achieved, acute exacerbations and chronic devastating complications are still encountered. There are several clinical and/or laboratory indicators alerting the physician to the possibility of a disease flare, the most important of which is the clinical evaluation of the patient.

### Laboratory tools

C-reactive protein (CRP), erythrocyte sedimentation rate (ESR) and leukocyte counts are usually elevated during active disease, but these tests are not specific because they are frequently elevated during infections as well [5,8,9]. In addition, normal levels do not always exclude the presence of active disease. The relation between disease activity and acute phase reactants such as ESR, CRP levels have been studied in adults. However it has been reported in only one pediatric study which showed a poor correlation between scoring indices (BVAS and PGA) and acute phase reactants (ESR and CRP) [28,29]. A urinalysis as well as liver and kidney function tests may indicate involvement of organ systems but they are likewise non-specific for active disease. They may be abnormal due to infection, co-morbidity, organ damage or drug toxicity.

ANCA- associated vasculitides (WG, MPA, CSS) are seen less commonly in children but they may result in significant renal or respiratory system damage. The introduction of widespread ANCA testing has assisted in the differential diagnosis of adult vasculitis. The accuracy of ANCA in determining the disease activity and distinguishing relapse is controversial [30]. In the adult literature, there is no consensus about the value of c-ANCA titers to assess/predict disease activity or progression [31-33]. In one study, only 24% of the patients had an increased level of c-ANCA before occurrence of a relapse [34]. In contrast, lower rates of c-ANCA positivity was reported in childhoodWG which is usually more localized in children. In the absence of clinical evidence of disease activity, the presence of the c-AnCA positivity is not sufficient unto itself to justify aggressive immunosuppressive therapy in children who may be adversely affected by the burden of these medications.

Anti-endothelial antibodies, von Willebrand factor and adhesion molecules are also studied in adults as markers of disease activity; however, they are nonspecific for disease activity as they reflect the endothelial impact of vasculitis [35-37]. These markers may be used more specifically with clinical assessment tools.

Although histopathological evaluation is the gold standard in making the diagnosis of vasculitis, its sensitivity and specificity differs between vasculitis subgroups. Furthermore, even in active renal disease, in which histopathological findings are almost always positive, it is impractical to re-biopsy patients for assessing disease activity.

### Radiologic evaluation

X-rays, ultrasonography, computerized tomography (CT), magnetic resonance imaging (MRI), positron emission tomography (PET) and conventional angiography are the main imaging techniques used in diagnosis of vasculitis and determination of organ damage [38-42]. MRI is a good non-invasive diagnostic and follow-up technique for TA [38]. In adults, CT is helpful for diagnosing ANCA-associated vasculitis, especially if there is upper or lower airway involvement; in children, it is very useful for detection of coronary aneurysms and stenosis in KD [41]. Ultrasound is an operator- dependent technique. Although CT and radiograph imaging are important, they have limitations due to contrast material and radiation exposure in children. MRI and ultrasound techniques may be of more practical use.

## Lessons From Adult Experience

Multicenter trials in adult rheumatology have provided evidence-based data for the treatment and management of adult vasculitides, under the structure of European Vaculitis Study Group (EUVAS) and other organizations such as the International Network for the Study of Systemic Vasculitis, the French Vasculitis Study Group and the Vasculitis Clinical Research Consortium (VCRC) [43-45]. These groups have independently conducted randomized controlled clinical trials (RCTs) using standardized clinical measurement scores. The results of these trials have had a significant impact on patient care in clinical practice [46-48].

Based on a proposal by EUVAS to the European Standing Committee for International clinical studies including therapeutics, a group of experts was formed including members of EUVAS and VCRC. The organizational progress of vasculitis investigators worldwide require parallel improvements in the ability to measure and record vasculitis activity in a uniform manner. The tools for the disease assessment and outcome measures were recommended and recently published by EULAR for conducting clinical studies and/or clinical trials in systemic vasculitis for adults (Table 2) [49].

**Table 2:** EULAR recommendations for disease assessment and outcome measures in systemic vasculitis for adults

Disease activity	Disease extent	Physician global assessment	Damage	Quality of life and generic health status measures
Birmingham Vasculitis Activity Score (BVAS Version 1)	The Disease Extent Index (DEI)		The Vasculitis Damage Index (VDI)	Short form-36 (SF-36)

Birmingham Vasculitis Activity Score (BVAS Version 2)				

Birmingham Vasculitis Activity Score (BVAS Version 3)				

Birmingham Vasculitis Activity Score for Wegener's granulomatosis (BVAS/WG).				

### Birmingham Vasculitis Activity Score (BVAS)

In 1994, the original version of BVAS was developed by a group of physicians dealing with vasculitis and validated by the same study. Later on, it was refined as BVAS v2 in 1997 [50,51]. The most recent version of BVAS (v3.0) has been refined from previous versions, based on extensive use in clinical trials and validated in a range of over 300 adults with vasculitis. The number of items was reduced to 56 from 66 in BVAS (v.3) [29]. BVAS aims to define the active disease requiring treatment. It assesses 9 organ systems (systemic, cutaneous, mucous membranes/eyes, ear/nose/throat, chest, cardiovascular, abdominal, renal and nervous system). Each individual item is recorded as being newly present or worsening within the last 4 weeks. The presence or absence of each item is recorded and each item has a weighted score. The total score of items give an objective measure for disease activity; the higher the score, the more active the vasculitis.

### Disease Extent Index

This instrument has been developed as a complementary measure to disease activity as measured by the BVAS. It has been validated for use in patients with Wegener's granulomatosis and partially validated in patients with hepatitis C virus-associated cryoglobulinemic vasculitis [52,53]. DEI was used in three RCTs and several open-label studies [53-56]. The DEI can be applied to patients with active disease as well as patients with partial or complete remission. The score sheet assesses 11 organ systems. Each system, excluding constitutional symptoms (score = 1), is weighted as 2 points if active vasculitis is present at the time of assessment. Each body system is scored only once, irrespective of the number of affected organ within the organ system or the severity of the respective manifestation. The final score for DEI may reach a maximum of 21. There is no distinction between new, worse or persistent disease. However, previous activity or damage is not counted. The DEI can complement the BVAS by showing whether a high BVAS score is due to multiorgan involvement or severe involvement of 1 organ.

### New insights in pediatric vasculitis

Pediatric rheumatologists have recently focused on developing outcome instruments in childhood vasculitis. Although BVAS has been validated in a large number of adult patients and accepted as the gold standard for assessment of disease activity, it may not be suitable for use in children with vasculitis, partly because some items are specific to adults. The items of BVAS could be re-evaluated according to the presence and frequency of clinical features of pediatric vasculitides, with some modification of items, deletion of some items and addition of new pediatric vasculitis specific items, as well as a change to the weighted scores. First and foremost is that the normal reference ranges in children are different from adults such as blood pressure, serum creatinine, amount of proteinuria, glomerular filtration rate or cut-off for significant weight loss. In addition, disease presentations as well as some clinical features and outcome are different in children compared to adults. Arthritis is more common in children with HSP and renal involvement usually increases with age [57]. WG is less frequent in children but subglottic stenosis is seen more commonly than in adults [9]. The other most apparent differences between adult and pediatric patients with vasculitis are developmental and growth issues, differences in functional, educational and social impact of longstanding disease and absence of adult co-morbidities. There are also distinctive drug pharmacokinetics and toxicity profiles in children.

An ideal assessment tool is the one that can differentiate active disease from damage or treatment complications and predict the functional outcome. Presently, there are efforts in place to develop a pediatric version of BVAS that is called pediatric vasculitis activity score (PVAS). The PVAS project, a newly developed tool for the evaluation of systemic vasculitis in children, was built on previous successful international collaboration of the PReS Vasculitis Working Party for childhood systemic vasculitis, in cooperation with members of Childhood Arthritis and Rheumatology Research Alliance (CARRA) from North America. Preliminary work from these investigators has derived a version of PVAS which has been validated in paper cases. Currently, PVAS is in a validation process on real pediatric vasculitis cases that will determine feasibility, reliability, internal consistency and tool responsiveness to change. The PVAS score will be correlated with routine clinical laboratory values such as ESR, CRP and, physician's global assessment of disease activity. The PVAS is meant to help physicians assess an individual patient's response to therapy and also serve as an outcome measures in clinical trials. Thus it might have a positive impact on the ability to research new therapies.

PVAS is designed to record new or worsening clinical features of active vasculitis that is attributable to the current state of the disease, after excluding other causes such as infection, hypertension, etc. PVAS includes a set of items that involves nine organ based systems similar to BVAS. The presence or absence of each weighted item gives a maximum score for each organ system. The sum of all nine organ systems determines the total score that shows the disease activity of each patient at the time of evaluation. If the patient is evaluated for the first time by the physician, all features of the disease are considered active/new irrespective of the time period. On subsequent evaluations, time has a more important role in formal distinguishing "new" active disease presentation from "persistent", low-grade or festering disease. Disease presentation is considered "active" if it has been newly present over the 4 weeks prior to the time of evaluation or whether it has worsened since the last evaluation and has been lasting for up to 3 months in total. The paper sheet includes the tick box: "No new/worse disease" and should be marked if there is no new/worse abnormality present in any of the systems. On this basis, the calculated score for PVAS will contain only values representing persistent disease. In other words, it represents no new disease and means all of the active features are persistent. If one item among the item list represents either new or worse disease, then the "No new/worse disease" box should not be checked.

After the PRINTO and PReS defined the classification criteria for primary systemic childhood vasculitis, they performed the first validation study in this field to use the original BVAS (v.3) and disease extent index (DEI) for the measurement of disease activity in childhood vasculitis [28]. In this study, the analysis data set included 796 patients with primary systemic vasculitides. There were 669 HSP, 80 c-PAN, 25 c-WG and 22 TA patients. The authors found a strong correlation between the BVAS v.3 and DEI (r_s _= 0.80) while correlation with PGA were moderate (r_s _= 0.49) and poor with CRP and ESR (r_s _= 0.34, r_s _= 0.31). Responsiveness was good for BVAS (SRM = 1.38) and DEI (SRM = 1.9). BVAS and DEI also seem sufficiently sensitive to detect the change in disease status over time. These two tools have been able to differentiate different vasculitis groups according to their results among childhood vasculitis. The correlation with the physician global assessment of disease activity was moderate in our series, similar to the data reported in the original BVAS paper by Luqmani et al. where the correlation was poor [50]. With this study, the authors demonstrated that the BVAS and DEI correlated poorly with ESR and CRP and the correlation did not improve when the analysis was repeated without the HSP patients that are known to have normal or low level of inflammation. On the other hand, there was a good correlation among these two instruments suggesting that the simplest DEI might be interchangeably used as an alternative measure to assess the disease activity in vasculitis, at least in children. The use of BVAS in children is hampered by the differences in both the spectrum of active disease manifestations as well as by some of the item definitions; this problem was overcome by the modification of the BVAS glossary to take into account pediatric specificities. Therefore the validation of the BVAS and DEI has to be considered the first step for the proper evaluation of disease activity.

### Patient-reported outcome measurements (PROMs)

The inclusion of PROMs is another standard research practice for rheumatic diseases in adults and children and provides a report of the status of a patient's health condition that comes directly from the patient. There are several examples of disease specific well-defined and reliable instruments in children with JIA such as health-related quality of life (HRQOL), the juvenile arthritis multi-dimensional assessment report (JAMAR), questionnaires for the evaluation of functional ability, and visual analog scales (VAS) for rating of child's overall well-being and intensity of pain. Recently a new tool named simple measure of the impact of lupus erythematosus in youngsters (SMILEY) has been developed for systemic lupus erythematosus (SLE) [58-61].

These instruments can be completed by the patients or parents on their behalf. Despite the development of instruments measuring disease extent, activity, and damage, there are no vasculitis-specific instruments measuring patient-estimated burdens of disease. Herlyn et al. recently published a study of PROMs among patients with vasculitis indicating that there were many manifestations of disease (fatigue, reduced energy level) that were quite important to patients but not measured with the outcome instruments currently included in clinical trials of vasculitis [62].

Patients' subjective experience should be a part of the outcome measurement process since they are the true experts about their disease. Therefore, the importance of the use of PROMs in medical outcome assessment (the OMERACT initiative) is also emphasized by the vasculitis clinical research community. Introduction of patient-based outcome assessments to childhood vasculitis will be one of the future directions.

### Damage

While dealing with disease activity, it is also important not to forget to define the amount of damage present in the patient. In 1997 the Vasculitis Damage Index (VDI) was defined for measuring the amount of damage [63]. It predicts the damage in 11 organ systems with irreversible pathology lasting for more than 3 months. Damage may be due to the disease or to the therapy. VDI has a role in predicting the outcome of the disease and probable mortality. A childhood version of the damage index is needed, because many items used in the adult version are not applicable to children such as the cut-off level to define hypertension. In addition, some aspects of damage are unique to children such as growth and development. A pediatric version of the VDI (PVDI) is being developed, as has previously been done with PVAS.

## Conclusion

While adult experience provides the backbone of most of the current practice in childhood vasculitis, there are some challenges in treatments and outcome measurement tools. The PReS has established a vasculitis study working group with international collaboration to cope with this problem. On behalf of the PReS and EULAR, PRINTO undertook a multinational, multicenter collaboration to validate BVAS and DEI as the activity measures. Because some of the items in BVAS were not considered appropriate for the pediatric population, the PReS Vasculitis Working Party developed the Pediatric Vasculitis Activity Score "PVAS" as a tool to assess disease activity in clinical trials based on the concept of the adult BVAS scoring system. After defining the outcome measures, defining the damage will be an absolute necessity in pediatric rheumatology. Therefore, the PVDI should be developed soon and be an important component of outcome measures to be used in clinical trials of vasculitis treatment in children. Lastly, it is clear that both patient-based outcome assessments and evaluation of the response to treatment during followup are very important as well and better tools for assessment of these parameters need to be developed soon. After the PVAS and PVDI are validated, these tools will be next on the agenda in pediatric rheumatology.

## List of abbreviations

PSV: The primary systemic vasculitides; HSP: Henoch-Schonlein purpura; KD: Kawasaki Disease; PAN: Polyarteritis nodosa; WG: Wegener's Granulomatosis; TA: Takayasu Arteritis; ESRD: End stage renal disease; MPA: Microscopic polyangiitis; CSS: Churg-Strauss syndrome; ACR: American College of Rheumatology; EULAR: European League Against Rheumatism; PRES: Pediatric Rheumatology European Society; PRINTO: Paediatric, Rheumatology International Trials Organisation; c-WG: Childhood Wegener's granulomatosis; c-PAN: Childhood polyarteritis nodosa; c-TA: Childhood Takayasu arteritis; JIA: Juvenile idiopathic arthritis; CRP: C-reactive protein; ESR: Erythrocyte sedimentation rate; ANCA: Antineutrophil cytoplasm antibody; CT: Computerized tomography; MRI: Magnetic resonance imaging; PET: Positron emission tomography; EUVAS: European Vaculitis Study Group; VCRC: Vasculitis Clinical Research Consortium; RCTs: Randomized controlled clinical trials; BVAS: Birmingham vasculitis activity score; PVAS: Pediatric vasculitis activity score; CARRA: Childhood Arthritis and Rheumatology Research Alliance; PGA: Physician global assessment; PROMs: Patient-reported outcome measurements; HRQOL: Health-related quality of life; JAMAR: Juvenile arthritis multidimensional assessment report; VAS: Visual analog scales; SMILEY: Simple measure of the impact of lupus erythematosus in youngsters; VDI: Vasculitis damage index; PVDI: Pediatric vasculitis damage index.

## Competing interests

The authors declare that they have no competing interests.

## Authors' contributions

ED is the main author and the corresponding author and has primarily designed and written the article. Coauthors RL, NAA, AK and SO have made substantial contributions to conception and design; have been involved in revising the manuscript and have given final approval of the version to be published. All authors read and approved the final manuscript.
